# Evaluation of the line probe assay for the rapid detection of bacterial meningitis pathogens in cerebrospinal fluid samples from children

**DOI:** 10.1186/s12866-016-0834-0

**Published:** 2017-01-11

**Authors:** Ahmet Soysal, Demet Gedikbasi Toprak, Salih Türkoğlu, Mustafa Bakir

**Affiliations:** 1 Division of Pediatric Infectious Diseases, Marmara University Medical Faculty Department of Pediatrics, Istanbul, Turkey; 2 University of Washington Seattle Children’s Hospital, Seattle, USA; 3 Anadolu Medical Center, Kocaeli, Turkey; 4 Division of Pediatric Infectious Diseases, Marmara University Pendik Training and Research Hospital Department of Pediatrics, Mimar Sinan Street, No:41, Fevzi Cakmak Mah. Ust Kaynarca, Pendik, Istanbul Turkey

**Keywords:** Line probe assay, Bacterial meningitis, Children, National surveillence

## Abstract

**Background:**

The aim of this study is to compare the diagnostic performance of the line probe assay (LPA) with conventional multiplex polymerase chain reaction (PCR) for *Streptococcus pneumoniae* as well as real-time PCR for *Neisseria meningitidis* and *Haemophilus influenzae* type b (Hib) in cerebrospinal fluid (CSF) samples from children during the multicenter national surveillance of bacterial meningitis between the years 2006 and 2009 in Turkey.

**Results:**

During the study period 1460 subjects were enrolled and among them 841 (57%) met the criteria for probable bacterial meningitis. The mean age of subjects was 51 ± 47 months (range, 1–212 months). We performed the line probe assay in 751 (89%) CSF samples of 841 probable bacterial meningitis cases, of whom 431 (57%) were negative, 127 (17%) were positive for *S. pneumoniae*, 53 (7%) were positive for *H. influenzae* type b, and 41 (5%) were positive for *N. meningitidis*. The LPA was positive in 19 of 23 (82%) *S. pneumoniae* samples, 4 of 6 (67%) *N. meningitidis* samples and 2 of 2 (100%) Hib samples in CSF culture-positive cases. The specificity of the LPA for all of *S. pneumoniae*, *H. influenzae* type b, and *N. meningitidis* was 88% (95% CI: 85–91%), when using the standard PCR as a reference. The specificity of LPA for each of *S. pneumoniae*, *H. influenzae* type b, and *N. meningitidis* was 93% (95% CI: 89–95%), 96% (95% CI: 94–98%), and 99% (95% CI: 97–99%), respectively. For all of *S. pneumoniae*, *H. influenzae* type b and *N. meningitidis* the sensitivity of the LPA was 76% (95% CI: 70–82%) and for each of *S. pneumoniae*, *H. influenzae* type b and *N. meningitidis* was 72% (95% CI:63–79%), 88% (95% CI: 73–95%), and 81% (95% CI:67–92%), respectively.

**Conclusions:**

The LPA assay can be used to detect common bacterial meningitis pathogens in CSF samples, but the assay requires further improvement.

## Background

National surveillances of bacterial meningitis can help identify the country-specific etiological agents and their serogroups/serotypes thus it can be provided information for implementation of vaccines in national immunization programs. The gold standard for the assessment of bacterial meningitis agents is the growth of bacteria in cerebrospinal fluid (CSF) culture. However, the unavailability of a microbiology laboratory, lack of quality control for media, suboptimal transport conditions or delays of the CSF samples and receiving oral or parenteral antibiotic treatment prior to lumbar puncture (L/P) can lead to low rates of CSF culture positivity. CSF culture positivity may be as low as 67 and 56% in patients who have received prior oral and parenteral antibiotics, respectively [1]. Because of these problems it may result in an underestimation of the disease burden and the potential impact of vaccination. Polymerase chain reaction (PCR), a non-culture based method, is an alternative approach for both the diagnosis and surveillance of bacterial meningitis with has high sensitivity and specificity. Currently, many experts have accepted either PCR or culture as the gold standard methods in developed and developing countries [2–5].

We have recently designed a line probe assay (LPA) [based on multiplex PCR followed by reverse hybridization using sequence-specific oligonucleotide probes (SSOP)] for the rapid detection of the major bacterial pathogens and their serotypes/serogroups that cause meningitis. Herein, we compare the diagnostic performance of the LPA with conventional multiplex PCR for *Streptococcus pneumoniae* as well as real-time PCR for *Neisseria meningitidis* and *Haemophilus influenzae* type b in CSF samples which were taken from children during the multicenter national surveillance of bacterial meningitis between the years 2006 and 2009 in Turkey.

## Methods

A prospective surveillance was performed in collaboration with the Department of Infectious Diseases, Ministry of Health between July 2006 and January 2009. A total of 37 hospitals located in 23 cities (representing 59% of the whole population of the country) across seven geographic regions participated. Ethical approval for the study was obtained from the ethical committee of the Marmara University School of Medicine. Written informed consent was obtained from the caregiver of each enrolled child. The surveillance study included children <18 years of age (excluding newborns) who were admitted to an emergency room and underwent lumbar puncture for suspected meningitis (based on signs and symptoms of meningitis, including fever, vomiting, headache, seizure, meningeal irritation, and impaired consciousness) and who met any of the following CSF laboratory criteria: [1] turbid CSF; [2] >99 leukocytes/mm^3^ in CSF; or [3] 10–99 leukocytes/mm^3^ in CSF with low CSF glucose (<40 mg/dL) and high CSF protein (>99 mg/dL) and no more than a few red blood cells in each mm^3^ of CSF [5]. The collected CSF specimens for PCR studies were stored at −20 °C until transported under cold-chain conditions to the Marmara University Hospital, Pediatric Infectious Diseases Research Laboratory. The samples were then stored at −80 °C until they were sent to the *Streptococcus* and *Meningococcus* Laboratories of the U.S. Centers for Diseases Control and Prevention, Atlanta for standard PCR analysis [6, 7]. The line probe assay was performed at the Marmara University Hospital Pediatric Infectious Diseases Research Laboratory using the rest of the CSF samples.

### DNA isolation

DNA was extracted from the CSF using a modification of the QIAamp DNA Mini kit (QIAGEN Inc., Valencia, CA, USA) method. All subsequent steps were performed as outlined in the QIAGEN DNA Mini protocol (for details, please see http://www.cdc.gov/ncidod/biotech/strep/pcr.htm) [8].

### Reference PCR

Pneumococcal detection was performed using an assay targeting the *lyt*A gene as previously described by Carvalho et al. [8]. A conventional sequential multiplex PCR, which is able to detect a total of 40 serotypes, was performed using 8 sequential reactions (see http://www.cdc.gov/ncidod/biotech/strep/pcr.htm for the latest updates). The primers which were used for this study were listed in our previous study [9]. Three triplex PCR assays were performed as previously described to detect the following: [1] the bacterial pathogens *N. meningitidis* (*ctrA*), *H. influenzae* (*hpd*) and *S. pneumoniae* (*lytA*) [10]; [2] the *N. meningitidis* serogroups A, X and W; and [3] the *N. meningitidis* serogroups B, C and Y [11]. Hib was identified by real-time PCR targeting the *bcs2* gene [10]. For all PCR assays, a specimen was considered positive if the Ct value was ≤35 and negative if the Ct value was >40. If a Ct value was >35 and ≤40, the specimen was diluted 10-fold and retested to determine whether PCR inhibitors were present. The specimen was considered positive if the Ct value of the diluted specimen was ≤35, equivocal if the Ct value was 36–40 and negative if >40. We accepted the reference PCR as the standard assay and compared the results of the line probe assay with the reference PCR results [6].

### Bacterial meningitis line probe assay

**Fig. 1:**
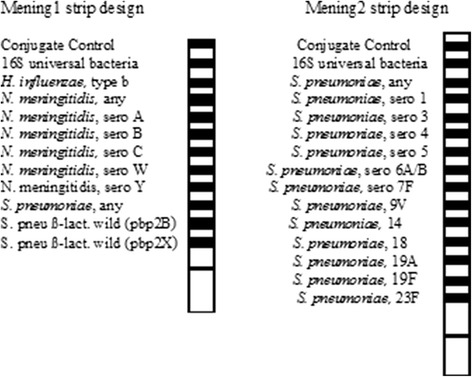
Schematic representation of the bacterial meningitis pathogens line probe assay

In this study, we used a line probe assay demo-kit produced by GenID GmbH, Straßberg, Germany, which is based on multiplex PCR followed by reverse hybridization using SSOP. A single Mening1 detection strip can identify universal bacterial 16 s DNA, three different meningitis-associated pathogens (*N. meningitidis*, Hib and *S. pneumoniae*), four serotypes of *N. meningitidis*, and the *S. pneumoniae* genes *pbp2B* and *pbp2X* (which are responsible for resistance to beta-lactam antibiotics). The Mening2 detection strip can identify universal bacterial 16 s DNA, *S. pneumoniae* and 13 different serotypes of *S. pneumoniae* (Fig. 1).

### Line probe assay procedure

LPA testing was performed following the manufacturer’s instructions (GenID GmbH, Straßberg, Germany). First, a multiplex PCR was performed using the Mening1 PN-Mix or the Mening2-PN-Mix using DNA isolated from a CSF sample. Following PCR, the respective biotinylated amplicons were characterized by a hybridization reaction with SSOPs for different pathogens and controls immobilized on a nitrocellulose membrane in a distinctive line format. During the hybridization, the denatured biotin-labelled amplicons bind to the gene probes attached to the strips. A highly specific washing procedure ensures the hybrids survive only if the sequence of the probe is 100% complementary to that of the amplified DNA. Streptavidin-coupled alkaline phosphatase binds to the biotinylated hybrids, and the complex is then detected by a color reaction after the addition of the substrate NBT/BCIP. Each test kit consists of 12 nitrocellulose strips coated with the following pathogen gene probes: Mening1 contains Hib, *N. meningitidis, S. pneumoniae* resistance genes, and Mening2 contains *S. pneumoniae* serotypes. The kit also contains denaturing reagent (solution 1), hybridization buffer (solution 2), stringent wash solution (solution 3), rinse solution (solution 4), conjugate solution, conjugate buffer (solution 5), substrate (solution 6) and an incubation tray.

Solutions 2 and 3 were pre-warmed to 47 °C, and all other solutions were pre-warmed to room temperature. Twenty microliters of solution 1 was added to each well, then 20 μl of PCR Mening1-PN-mixture was added to the denaturing reagent for the Mening1 strip or 20 μl of PCR Mening2-PN-mixture was added for the Mening2 strip. The samples were then mixed well by pipette and incubated for 5 min at room temperature. Then, 1 μl of pre-warmed solution 2 was added to each well, and the strips were transferred with forceps from the tube into the incubation tray. The strips were completely covered by liquid with the printed number facing upwards. The tray was incubated for 30 min at 47 °C in a shaking water bath. The hybridization buffer was then completely discarded, and the strips were washed twice for 1 min each with 1 ml of pre-warmed solution 3. Then, 1 ml of solution 3 was added to each strip, and the strip was incubated for 15 min at 47 °C in the shaking water bath. Solution 3 was removed by tapping, and the strips were washed twice for 1 min with 1 ml of solution 4. Then, 1 ml of solution 5 diluted 1:1000 was added to each strip, and the strips were incubated for 30 min on a horizontal shaker at room temperature. The conjugate was then removed, and each strip was washed 3 times with 1 ml of solution 4 for 1 min on a horizontal shaker. Then, 1 ml of solution 6 was added to each strip, and the strips were incubated up to 10 min, depending on the intensity of color development. After the control lines appeared and before the background staining emerged by washing twice with 1 ml of distilled water the conjugate reaction was stopped. Then, the strips were transferred by forceps from the wells to absorbent paper and were dried protecting from light. After drying, the strips were placed on a background sheet of white paper, and the reaction zone was marked with the supplied template.

### Statistical analysis

CDC PCR accepted as a reference test and the sensitivity of Line Probe Assay for each pathogen was calculated as: true positive LPA result/true LPA positive result + false negative LPA result. The specificity of LPA for each pathogen was calculated as true negative LPA result/false positive LPA result + true negative LPA result. The sensitivity and specificity with 95% confidence interval of LPA for any bacterial pathogen and each pathogen were calculated using using MedCalc easy to use statistical software (https://www.medcalc.org/calc/diagnostic_test.php).

## Results

Among 1460 subjects, 841 (57%) met the criteria for probable bacterial meningitis. The mean age of the subjects was 51 ± 47 months (range,1–212 months), and 550 (65%) subjects were male. Oral or parenteral antibiotic treatment prior to lumbar puncture was reported in a significant proportion of patients [*n* = 435 (40%)]. CSF cultures were positive for *S. pneumoniae*, *N. meningitidis,* and *H. influenaze* type b in 23, 6, and 2 patients, respectively.

### Line probe assay

Among the 841 probable bacterial meningitis cases, we were able to perform the line probe assay in 751 (89%) CSF samples; among these samples 431 (57%) were negative, 127 (17%) were positive for *S. pneumoniae*, 53 (7%) were positive for *H. influenzae* type b, 41 (5%) were positive for *N. meningitidis*, 2 were positive for both *S. pneumoniae* and *H. influenzae* type b, and 2 were positive for both *H. influenzae* type b and *N. meningitidis*. A strip band for universal bacterial 16 s DNA was detected in 95 (13%) CSF samples. We also detected strip bands in 19 CSF samples, 12 of which were for *pb2b* and *pb2x* [displayed a band for *S. pneumoniae* (*n* = 8), 16 s DNA (*n* = 2), both *H. influenzae* type b and *N. meningitidis* (*n* = 1) and was negative (*n* = 1)], 6 of them were for *pb2b* [had a band for *S. pneumoniae* (*n* = 5) and *H. influenzae* type b (*n* = 1)] and 1 for *pb2x* [also had a band 16SDNA (*n* = 1)].

In the CSF culture-positive cases, the LPA was positive in 19 of 23 (82%) *S. pneumoniae* samples, 4 of 6 (67%) *N. meningitidis* samples and 2 of 2 (100%) *H. influenzae* type b samples.

### Comparison of the LPA with the reference PCR

The results of LAP vs. standard PCR are shown in Table 1. The specificity of the LPA for all tested samples was 88% (95% CI: 85–91%), using the standard PCR as a reference. The specificity of LAP for *S. pneumoniae*, *H. influenzae* type b, and *N. meningitidis* was 93% (95% CI: 85–91%), 96% (95% CI: 94–98%) and 99% (95% CI: 97–99%), respectively. The sensitivity of the LPA for *S. pneumoniae*, *H. influenzae* type b and *N. meningitidis* combined was 76% (95% CI: 70–82%), while for each pathogens individually it was 72% (95% CI:63–79%), 88% (95% CI: 73–95%), and 81% (95% CI:67–92%) for *S. pneumoniae*, *H. influenzae* type b and *N. meningitidis* respectively. Of the 3 PCR-positive samples for non-serotype b *H. influenzae*, both samples were negative by LPA. Among the 19 samples with equivocal PCR results (Ct values of 36–40) for *S. pneumoniae lytA*, we performed the LPA in 18 samples, 12 of them were negative, five were positive for *S. pneumoniae* and one was positive for universal bacterial 16 s DNA. Among the six samples with equivocal PCR results for *H. influenzae hpd*, LPA was negative for all of four sample we tested. Among the seven samples with equivocal PCR results for *N. meningitidis ctrA,* LPA was negative for both of two sample we tested. In total, of the 32 samples with equivocal PCR results, we were able to perform the LPA in 22 samples, 5 of them (23%) were positive.

**Table 1:** Comparison of the Line Probe Assay with the Reference PCR for bacterial meningitis in CSF samples

LPA results	Reference PCR results (n)								
	Negative	Sp	Hib	Men	Hi	*lytA* Eq	*hpd* Eq	*ctrA* Eq	Total
Negative	380	24	1	6	2	12	4	2	431
Sp	30	92	0	0	0	5	0	0	127
Hib	15	3	35	0	0	0	0	0	53
Men	4	2	0	35	0	0	0	0	41
16S DNA	92	2	0	0	0	1	0	0	95
Double banding Sp or Hib	0	0	1	1	0	0	0	0	2
Double banding Hib or Men	1	1	0	0	0	0	0	0	2
Not done	73	7	3	5	1	1	0	0	90
Total	595	131	40	47	3	19	4	2	841

*LPA* line probe assay, *Sp Streptococcus pneumoniae*, *Hib H. influenzae* type b, *Men N. meningitidis*, *Hi* non-typeable *H. influenzae*, *lytA Eq* equivocal for *ltyA*, *hpd Eq* equivocal for *hpd, ctrA Eq* equivocal for *ctrA*

#### *S. pneumoniae* serotype comparison

Because the LPA was designed to detect only 13 serotypes of *S. pneumoniae*, we compared the line probe assay to standard PCR with respect to these serotypes. The results from 53 samples tested by LPA were concordant with 41 samples (77%) tested by PCR. The test positivity of the line probe assay for serotypes 4, 6A/6B, 9 V/9A, 14, 18, 19 F, 23 F, 3, 19A, 1, 5 and 7 F was 100% (2/2), 80% (4/5), 50% (1/2), 100% (4/4), 60% (3/5), 72% (8/11), 100% (6/6), 100% (1/1), 0% (0/4), 70% (7/10), 100% (5/5) and 0 (*n* = 0), respectively.

#### *N. meningitidis* serogroup comparison

The results from 18 samples tested by LPA were concordant with 35 samples (51%) tested by the standard PCR. The test positivity of the LPA for serogroups A, B, C and W was 100% (1/1), 38% (9/24), 100% (2/2) and 0% (0/1), respectively.

## Discussion

This is the first study to report the performance of the LPA in the detection of common bacterial pathogens, including *S. pneumoniae*, *N. meningitidis* and *H. influenzae* type b and their serotypes/serogroups in CSF clinical samples which were taken from children during a national surveillance of bacterial meningitis. LAPs are a group of novel DNA strip-based tests that use nucleic acid amplification techniques and reverse hybridization methods for the rapid detection of genes which are specific for certain groups of bacteria or mutations associated with drug resistance. In our demo-kit, the following genes were used for detection: the *ctrA* gene for detection of any *N. meningitidis* serotype, *sacB* for *N. meningitidis* serogroup A, *siaD* for *N. meningitidis* serogroup B, *siaD* for *N. meningitidis* serogroup C and *synF/G* for *N. meningitidis* serogroup W/Y; the *piuA* gene for any *S. pneumoniae* and *cps* genes for serotypes of *S. pneumoniae*; and the *hpd* gene for *H. influenzae*. In this study, we compared the line probe assay with reference PCR assays used by CDC that is currently used for detection and serotyping of bacterial meningitidis-associated pathogens [9–12]. This surveillance of bacterial meningitis represented approximately 60% of the population in Turkey before the introduction of pneumococcal and meningococcal conjugate vaccines [13]. PCR is well known to be a more sensitive method than culture in patients with previous antibiotic use prior to lumbar puncture In this study, 40% of the patients had a history of oral or parenteral antibiotic usage before L/P, and only 6% of these patients displayed CSF culture positivity; these results underestimate the presence of common bacterial pathogens and mislead the decisions regarding to which vaccine should be implemented in a national immunization program and the potential effectiveness of the chosen vaccine. In our study, although limited number of the CSF culture-positive cases were identified (*n* = 31), the positivity of the LPA was 83% for *S. pneumoniae*, 67% for *N. meningitidis,* and 100% for *H. influenzae* type b, respectively, in culture-positive samples. In contrast, when we compared the LPA results with the CDC reference PCR, the specificity was 88%. The specificity of LPA for each pathogens for *S. pneumoniae*, *H. influenzae* type b, and *N. meningitidis* was 93, 96, and 99%, respectively. The sensitivity of the line probe assay for all of *S. pneumoniae*, *H. influenzae* type b, and *N. meningitidis* was 76, and for each *S. pneumoniae*, *H. influenzae* type b, and *N. meningitidis* it was 72, 88 and 81%, respectively. The detection of *S. pneumoniae*, *N. meningitidis,* and *H. influenzae* type b by LPA was generally acceptable with slightly lower overall specificity, although it has a comparable specificity for each pathogens. Overall sensitivity and specificity of LPA for detection of *S. pneumoniae* is lower than expected, but LPA has a better sensitivity for detection of *H. influenzae* type b and *N. meningitidis*. The lower sensitivity of LPA detecting *S. pneumoniae* may be explained by usage of large number of (*n* = 10) primer pairs in strip 1. We think that use of a lower number of primer pairs including only a conjugate control, the universal bacterial 16S gene, *H. influenzae* type b, *N. meningitidis* (any serotype), and *S. pneumoniae* (any serotype) may improve sensitivity of LPA. This modification may eliminate the double banding for all bacteria and the penicillin-resistance gene of *S. pneumoniae*. Secondly, *ctrA* and *hpd* genes which are used for detection of *N. meningitidis* and *H. influenzae* type b, respectively in LPA are also used in CDC standard PCR. Hence, we achived higher than %95 sensitivity and specificity rate for both pathogen. On the other hand, for detection of *S.pneumoniae, piuA* gene was used in LPA versus *lytA* gene was used in CDC standard PCR. Usage of different genes possibily lead to lower sensitivity of LPA for detection of *S. pneumoniae*. In this study, we could not assess the antibiotic resistance of isolates because of the lack of microbiology laboratory infrastructure for antibiotic susceptibility tests in many of the participated hospitals and very low rate of culture positivity. The LPA also provides us an opportunity to simultaneously determine the serogroups of *N. meningitidis* and *S. pneumoniae* at the same reaction. Among the *N. meningitidis* serogroups, the lowest positivity was achieved for serogroup B (38%) which is the considerable serogroup in Europe and within our country [13, 14]. For serogroups A, C, W and Y, we could not make a conclusion because of the low numbers of each serogroup. For *S. pneumoniae* serotype determination was possible in a sufficient number of patients to conclude that the positivity was 80, 100, 80, 72, 100, 70 and 100% for st6A/6B, st14, st18, st19F, st23F, st1 and st5, respectively. Unfortunately, the LPA performance was poor for serotype 19A, which might be due to the insufficient sensitivity of the probe used for this serotype. The gold-standard serotyping method for *S. pneumoniae* is the Quellung reaction which was described in the early 1900s and is based on testing colonies with a set of antisera and visualising the bacteria under a microscope [15]. It is laborious and requires a complete set of type-specific antisera, and is therefore mainly performed by reference laboratories. Alternate serotyping methods have been developed, but few data formally comparing the performance of these methods to the gold-standard Quellung reaction, or to each other, are available. Satzke et al. evaluated the best pneumococcal serotyping methods for *S. pneumoniae* carriage studies in a large international multi-center study [15]. They invastigated 20 pneumococcal serotyping methods including direct multiplex PCR, culture mPCR, direct mPCR/reverse line blot, culture restriction fragment length polymorphism, culture mPCR and microarray, culture microarray and direct microarray. They conclude that most of these methods were able to detect the dominant serotype in a sample, but many of them performed poorly in detecting the minor serotype populations.

A commercially available LPA for the detection of common bacterial meningitis pathogens does not currently exist, but several LPAs are commercially offered to detect drug resistance in *Mycobacterium tuberculosis* strains [16, 17]. In addition to detecting *M. tuberculosis* and its resistance to drugs, the line probe assay has also recently been used to detect extended-spectrum beta-lactamase and KPC carbapenemase genes in Enterobacteriacea [18].

## Conclusions

The line probe assays have the following advantages: a) the assays are easy to perform; b) the assays do not require special equipment, except for a simple thermal cycler; c) results are obtained within 1 day; and d) the assays can simultaneously detect 3 common bacterial pathogens and their serogroups/serotypes. However the primary limitation of the present study is the low number of gold standard CSF bacterial culture-positive cases included as a result of antibiotic usage prior to L/P in nearly half of our patients. Secondly, we compared the demo-kit LPA with standard PCR, but the genes which were used to detect the *S.pneumoniae* serotypes and *H. influenzae* type b and *N.meningitidis* serogroups in our kit were different than those used in the standard PCR, that caused difficulty in making precise conclusions. However this is the first clinical study evaluating LPA in national bacterial surveillance, the results of this study are prosperous. In conclusion, the LPA assay can be used to detect common meningitis bacterial pathogens in CSF samples, but the assay requires further improvement, especially in the strip design (including the probes used).

## Abbreviations

CSF: Cerebrospinal fluid
DNA: Deoxyribonucleic acid
Hib: *Haemophilus influenzae* type b
L/P: Lumbar puncture
LPA: Line probe assay
PCR: Polymerase chain reaction
SSOP: Sequence-specific oligonucleotide probes

## References

[1] Kanegaye JT, Soliemanzadeh P, Bradley JS (2001). Lumbar puncture in pediatric bacterial meningitis: defining the time interval for recovery of cerebrospinal fluid pathogens after parenteral antibiotic pretreatment. Pediatrics.

[2] Chanteau S, Sidikou F, Djibo S, Moussa A, Mindadou H, Boisier P (2006). Scaling up of PCR- based surveillance of bacterial meningitis in the African meningitis belt: indisputable benefits of multiplex PCR assay in Niger. Trans R Soc Trop Med Hyg.

[3] Chatelet I, Traore Y, Gessner B, Antignac A, Njanpop-Lafourcade B, Ouedraogo M (2005). Bacterial meningitis in Burkina Faso: surveillance using field-based polymerase chain reaction testing. Clin Infect Dis.

[4] Robbins J, Schneerson R, Gotschlich E (2005). Surveillance for bacterial meningitis by means of polymerase chain reaction. Clin Infect Dis.

[5] Won H, Yang S, Gaydos C (2012). A broad range assay for rapid detection and etiologic characterization of bacterial meningitis: performance testing in samples from sub-Sahara. Diagn Microbiol Infect Dis.

[6] Pimenta FC, Roundtree A, Soysal A (2013). Sequential triplex real-time PCR assay for detecting 21 pneumococcal capsular serotypes that account for a high global disease burden. J Clin Microbiol.

[7] Wang X, Mair R, Hatcher C (2011). Detection of bacterial pathogens in Mongolia meningitis surveillance with a new real-time PCR assay to detect Haemophilus influenzae. Int J Med Microbiol.

[8] Carvalho Mda G, Tondella ML, McCaustland K (2007). Evaluation and improvement of real-time PCR assays targeting lytA, ply, and psaA genes for detection of pneumococcal DNA. J Clin Microbiol.

[9] Da Gloria Carvalho M, Pimenta FC, Jackson D (2010). Revisiting pneumococcal carriage by use of broth enrichment and PCR techniques for enhanced detection of carriage and serotypes. J Clin Microbiol.

[10] Tzanakaki G, Tsopanomichalou M, Kesanopoulos K (2005). Simultaneous single-tube PCR assay for the detection of, Haemophilus influenzae type b and *Streptococcus pneumoniae*. Clin Microbiol Infect.

[11] Wang X, Theodore MJ, Mair R (2012). Clinical validation of multiplex realtime PCR assays for detection of bacterial meningitis pathogens. J Clin Microbiol.

[12] Dolan J, Satterfeild D, Hatcher C, et al. Real-time PCR assays for the detection of H. influenzae serotypes a, b, and f and sequencing of the capsule biosynthesis operons of serotypes c and d. In: Proceedings of the 110th ASM General Meeting 2010, San Diego

[13] Toprak D, Soysal A, Torunoğlu MA (2014). PCR-based national bacterial meningitis surveillance in Turkey: years 2006 to 2009. Pediatr Infect Dis J.

[14] Sridhar S, Greenwood B, Head C (2015). Global incidence of serogroup B invasive meningococcal disease: a systematic review. Lancet Infect Dis.

[15] Satzke C, Dunne EM, Porter BD, Klugman KP, Mulholland EK, PneuCarriage project group (2015). The PneuCarriage Project: A Multi-Centre Comparative Study to Identify the Best Serotyping Methods for Examining Pneumococcal Carriage in Vaccine Evaluation Studies. PLoS Med.

[16] World Health Organization. Policy statement. Molecular line probe assays for rapid screening of patients at risk of multidrug-resistant tuberculosis (MDR-TB). Geneva; 2008.

[17] Molina-Moya B, Lacoma A, Prat C, Diaz J, Dudnyk A, Haba L, Maldonado J, Samper S, Ruiz-Manzano J, Ausina V, Dominguez J. AID TB resistance line probe assay for rapid detection of resistant Mycobacterium tuberculosis in clinical samples. J Infect. 2014;4453(14):00305–3. doi: 10.1016/j.jinf.2014.09.010. [Epub ahead of print]) 10.1016/j.jinf.2014.09.010 25305498

[18] Bloomberg GV, Polsfuss S, Meyer V, Böttger EC, Hombach M (2014). Evaluation of the AID ESBL line probe assay for rapid detection of extended-spectrum b-lactamase (ESBL) and KPC carbapenemase genes in Enterebacteriacea. J Antimicrob Chemother.

